# Combined Anti-PD-1 and Anti-CTLA-4 Treatment in Stage IV Melanoma Patients: A Bicentric Analysis of Real-World Data and a Modern Treatment Scenario Proving Lactate Dehydrogenase’s Usefulness

**DOI:** 10.3390/diagnostics14060654

**Published:** 2024-03-20

**Authors:** Alexandru Dorin Adrian Silași, Anna Carolin Sievert, Paul Danciu, Andrei Vlad Lefter, Vlad Adrian Afrasanie, Daniel Sur

**Affiliations:** 1 Faculty of Medicine, Iuliu Hațieganu, University of Medicine and Pharmacy, 400347 Cluj-Napoca, Romania; anna.sievert@gmx.de (A.C.S.); daniel.sur@umfcluj.ro (D.S.); 2 Department of Oncology, Bistrița County Emergency Clinical Hospital, 420094 Bistrița, Romania; 3 Department of Oncology, The Oncology Institute “Prof. Dr. Ion Chiricuţă”, 400015 Cluj-Napoca, Romania; sirpauldanciu@gmail.com; 4 Faculty of Medicine, University of Medicine and Pharmacy “Grigore T. Popa”, 700483 Iași, Romania; andrei13vlad@yahoo.com (A.V.L.);; 5 Department of Oncology, The Regional Institute of Oncology, 700483 Iași, Romania

**Keywords:** melanoma, metastasis, overall survival, progression-free survival, side effects, nivolumab, ipilimumab, anti-programmed death-1 antibodies, anti-cytotoxic T-lymphocyte antigen-4 antibodies

## Abstract

Background: This retrospective study evaluates patients with stage IV melanoma treated with nivolumab and ipilimumab combination therapy from two regional oncology centers in Romania from the year 2019 to the end of 2022. Methods: The data were analyzed in SAS for Windows, V9.4. LDH means were stratified by the number of metastatic sites before treatment and compared using an independent sample T-test. The survival curves were estimated using the Kaplan–Meier method, and the survival distributions were compared with the log-rank test. The effects of the main clinical and pathological variables on OS and PFS were investigated with Cox regression. Results: The LDH mean for patients with three or more metastases before treatment was significantly higher than that for patients with only one metastatic site. The Kaplan–Meier curve of OS in all evaluable patients enrolled in the study resulted in a median OS of 346 days (95% CI: 150) and a median PFS of 211 days (95% CI: 113–430). A total of 45.3% of the patients experienced adverse events during the nivolumab + ipilimumab treatment, with some of them having multiple organ systems involved. Discussion: The OS values were lower than those reported in approved clinical trials, but the results show a marked improvement when compared to the results obtained by chemotherapy regimens previously used in these scenarios. Conclusion: This study provides real-world insights into the survival data and safety profiles of combination therapy with anti-PD-1 antibodies and anti-CTLA-4 antibodies.

## 1. Introduction

Melanoma of the skin is an important public health-related issue. In Romania, Global Cancer Statistics 2020 estimates the 5-year prevalence at 25.04/100,000 inhabitants and places it as the 20th cancer based on the number of new cases [1]. The landscape of skin melanoma treatment has been forever changed with the introduction of anti-programmed death-1 antibodies, anti-cytotoxic T-lymphocyte antigen-4 antibodies, and finally, targeted therapies such as BRAF and MEK inhibitors [2]. 

In the context of a pathology with such an impact on public health [3], a serum tumor marker that can help in the diagnostic process can prove invaluable. While being one of the first markers that proved to be useful in the treatment of skin melanoma, lactate dehydrogenase (LDH) [4] proves its usefulness time and time again as part of the initial assessment when facing a patient with skin melanoma. LDH is an enzyme responsible for the conversion of pyruvate to lactate [4]. It can be found in the cytoplasm of melanoma cells that replicate and thrive through oxygen-low dependence mechanisms, such as anaerobic pathways [5,6]. The upregulation of this cytoplasmatic enzyme helps the cancer cells adapt to the low-oxygen environment that is created by the ever-expanding clone of cancer cells, with demand outpacing the supply of oxygenated blood supplied by the neoangiogenic blood vessels [5]. The serum LDH levels rise when a large number of cells with high intracytoplasmic LDH levels spill their content into the bloodstream when cell death occurs. While in past research it was tough that high LDH levels were only associated with liver metastasis, newer data show this assumption to be false [7,8]. Now, the LDH level is associated with a multi-site skin melanoma spread [9] and has diagnostic, prognostic, and predictive values [8,10].

The conceptual shift in the therapeutic approach with regards to patients with stage IV melanoma took place with the release of the CheckMate studies, such as CheckMate 067 [11], which concluded that combination therapy with nivolumab and ipilimumab, or nivolumab alone in advanced melanoma, yielded a better OS than ipilimumab alone. In another study, CheckMate 066 [12], significant survival benefits, favorable safety profiles, and a favorable quality of life were achieved when comparing nivolumab to dacarbazine. Subsequent changes in the guidelines placed checkpoint inhibitors and targeted therapies at the forefront of advanced melanoma treatment. These changes came in a context in which patients were treated beforehand in an adjuvant or metastatic setting with therapies such as interferon alfa-2b or different regimens of chemotherapy such as dacarbazine, temozolomide, or platinum doublets [13,14]. 

This study retrospectively evaluates stage IV melanoma patients treated with nivolumab and ipilimumab combination therapy with regards to survival data, incidence, and severity of side effects based on real-world data collected from The Oncology Institute “Prof. Dr. Ion Chiricuță” Cluj-Napoca, Romania, and The Regional Institute of Oncology, Iași, Romania, between the years of 2019 and 2022. While this type of novel agent has been adopted in day-to-day practice in Romania, local real-world data (RWD) regarding these types of therapies have not been published to the knowledge of the author. RWD can prove an invaluable source of information as some clinical scenarios that are encountered frequently in clinical practice have not been explored in approved clinical trials. Therefore, patients with characteristics omitted in such trials, for example, patients with ECOG 2 performance status and patients with brain metastasis, can yield different results from these therapies than those reported in clinical trials. Also, the subgroup analyses provide invaluable information about patients’ characteristics, disease-related particularities, and serological markers that can have predictive, prognostic, and potentially even diagnostic value. 

## 2. Materials and Methods

### 2.1. Patients and Study Design 

We retrospectively enrolled patients with stage IV melanoma who underwent combined treatment with anti-programmed death-1 antibodies—nivolumab—and anti-cytotoxic T-lymphocyte antigen-4 antibodies—ipilimumab. The patients were enrolled in the two centers from Romania between January 2019 and the end of December 2022. Inclusion criteria were a histologically confirmed diagnosis of melanoma, tumor stage IV according to AJCC 2018 (8th edition), and eligibility for nivolumab–ipilimumab treatment according to national guidelines.

Medical data were collected from the local database of each institution, and all patients who underwent Nivolumab–ipilimumab combined treatment between the years mentioned above was investigated. After verifying the inclusion criteria, we identified a total of 57 patients. From the identified subjects, we excluded 4 patients due to incomplete medical data that prevented correct appreciation of OS, PFA, or specific data used in subgroup analyses.

PFS was defined as the time from the start of therapy to the date of the first progression of the disease, or death of the patient. For patients without disease progression at the time of analysis, PFS was censored on the date of last patient contact. OS was defined as the time from the start of therapy until the patient decease date, for patients alive at the date of analysis the values were censored. Adverse effects (AEs) were identified and rated by the center in which the patient underwent treatment by the Common Terminology Criteria for Adverse Events, Version 5 (CTCAE). Subgroup analyses included gender, BRAF-mutation status, LDH levels, previous therapies, and completion of the ipilimumab sequence. The study was carried out based on the approval of the Ethics Committees of the Oncology Institute “Prof. Dr. Ion Chiricuţă”, Cluj, Romania. All patients gave their consent to the Declaration of Helsinki.

### 2.2. Statistical Analyses

The data were analyzed in SAS for Windows, V9.4 (SAS Inc., Cary, NC, USA). LDH means were stratified by the number of metastatic sites before treatment and compared using an independent sample *t*-Test. OS was defined as the time from the start of the nivolumab + ipilimumab sequence of treatment until the date of death from any cause. The patients alive at the time of analysis were censored. PFS was defined as the time between the start of the nivolumab + ipilimumab sequence of treatment until the first record of disease progression or death from any cause. The survival curves were estimated using the Kaplan–Meier method, and survival distributions were compared with the log rank test. The effects of the main clinical and pathological variables on OS and PFS were investigated with Cox regression.

## 3. Results

### 3.1. Patients

A total of 53 patients receiving nivolumab + ipilimumab in a metastatic setting were included from the two centers in Romania. The median age of the patients was 54.1 ranging between 23 and 77 years old. 

The data were collected from the independent databases of the Regional Institute of Oncology, Iași, and The Oncology Institute “Prof. Dr. Ion Chiricuţă” Cluj-Napoca; after the exclusion process (incomplete data, patients receiving the same regimen in a different setting, etc.), a total of 18 patients from the Regional Institute of Oncology, Iași, were included in the final database and the rest were included from The Oncology Institute “Prof. Dr. Ion Chiricuţă” Cluj-Napoca. While each institution compiled an initial database with the patients presenting the characteristics mentioned in the Section 2, an independent final review and selection of the included subjects were performed to double-check that the included individuals met the inclusion criteria.

The sex distribution was homogenous with 49.1% female and 50.9% male patients. ECOG status ranged between 0 and 2, with most patients (62.3%) having ECOG 1, 22.6% having ECOG 0, and 15.1% ECOG 2. LDH levels were within normal ranges at the start of the treatment sequence for 56.6% of the patients. BRAF mutation status was evenly matched, with 43.4% mutated and 47.2% wild type. 

With regards to the distribution of metastasis at the start of combined anti-PD-1 and anti-CTLA-4 antibodies, non-regional lymph node metastases were present in 30 (56.6%) patients. Skin, and other soft tissue metastases, such as muscle or other visceral organs not mentioned, e.g., spleen, were present in 19 patients (35.8%). Bone metastases were present in 18.9% of patients. While the most frequent visceral sites of disease were lung metastases present in 50.9% of subjects and hepatic metastases present in 34% of subjects there was also an important percentage of patients with central nervous system (CNS) metastases at the start of therapy, 9 (17%), as seen in Table 1. The most frequent sites of distant disease progression observed during combined treatment were lung metastases closely followed by CNS and bone metastases while local progression occurred in only 1,9% of patients, as seen in Table 1. 

### 3.2. LDH Levels as a Potential Diagnostic Tool for Patients with Skin Melanoma in a High-Burden Disease Scenario

Analyzing mean LDH levels stratified by the number of metastatic sites showed a progressive growth of mean serum LDH levels as a higher number of metastatic sites were affected. For patients with one metastatic site, regardless of the organ involved (this includes non-CNS visceral sites of metastasis, nonregional lymph node, CNS, distant skin metastasis, and other soft tissue metastasis), there was a mean LDH level (U/L) (n = 10) 196.2 (116–399) (SD = 78.72). For patients with two metastatic sites (n = 19), there was a mean LDH level of 307.36 (144–1643) (SD = 338.19); and for patients with three or more metastatic sites (n = 17), there was a mean LDH level of 357.23 (99–1159) (SD = 264.04). 

A comparison between the group’s means was performed using an independent samples T-Test, when comparing one metastatic site (M = 196.2, SD = 78.72) vs. two metastatic sites (M = 307.36, SD = 338.19), statistical significance was not achieved, t (21.44) = 1.36, *p* = 0.187. The comparison between the group with three or more metastatic sites (M = 357.23, SD = 264.04) with the group with only one metastatic site achieved statistical significance, t (20.37) = 2.34, *p* = 0.02. Based on these results, LDH levels have a high chance to be elevated in patients that present with three or more sites of metastatic disease.

### 3.3. Treatment and Survival

**Figure 1:**
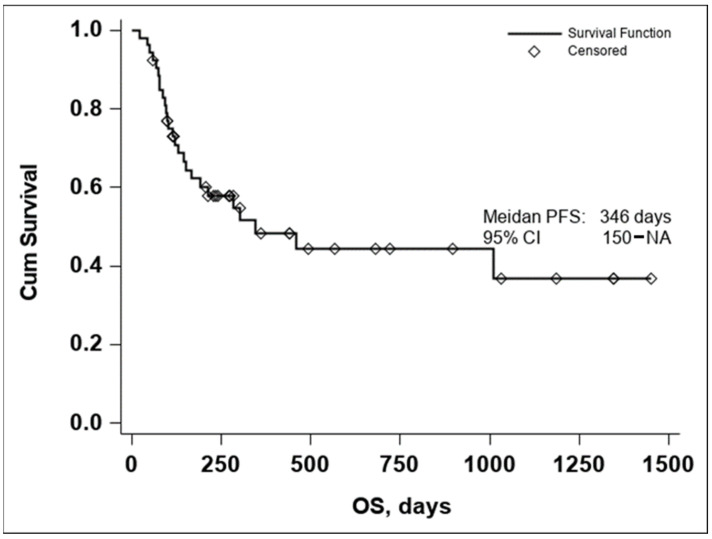
Kaplan–Meier curve of OS in all evaluable patients enrolled in the study (n = 53).

The Kaplan–Meier curve of OS in all evaluable patients enrolled in the study resulted in a median OS of 346 days, Figure 1. 

Due to the small number of events in the investigated group, the upper confidence interval of the Kaplan–Meier estimator for OS is not available, thus the 95% CI for OS was 150–NA.

The lower median OS in comparison to Checkmate 067 [2] is to be expected due to the different inclusion criteria. While the Checkmate study included previously untreated, unresectable, stage III or stage IV melanoma with a status performance of 0 or 1, in our study only stage IV patients were included, and a significant number having central nervous system, lung, and hepatic involvement, as seen in Table 1. The differences between the two populations are to be further detailed in the Section 4. 

**Figure 2:**
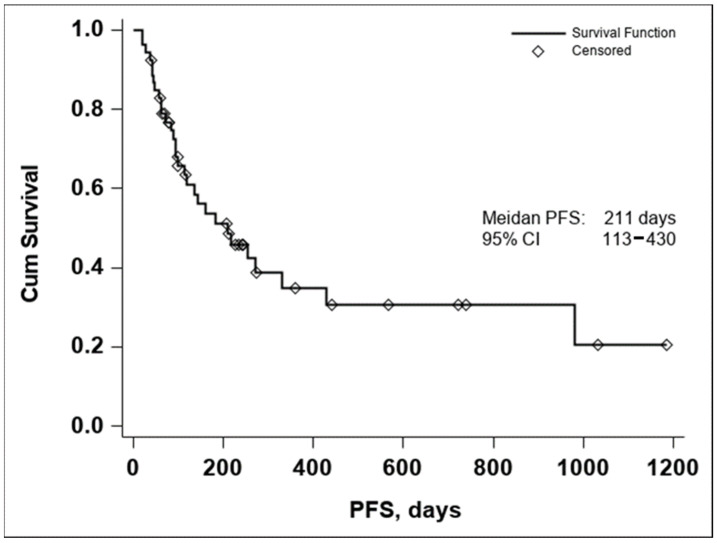
Kaplan–Meier curve of PFS in all evaluable patients enrolled in the study (n = 53).

The median PFS was 211 days with a 95% CI (113–430), as seen in Figure 2. In both the Kaplan–Meier curves, there can be noticed an aggregate of events occurring around the 250-day mark for OS and the 200-day mark for PFS, with an important number of subjects maintaining the response to treatment if no disease progression or death occurred until the aforementioned time marks. In conclusion, these data seem to indicate that if a response to treatment is observed past the 250-day mark, a high chance of maintaining this response for an extended period exists.

### 3.4. Adverse Events

In total, 45.3% of the patients experienced adverse events during the nivolumab + ipilimumab treatment with some of them having multiple organ systems involved, as shown in Table 2. AEs above ≥3 were reported in 26.4% of patients, with one death due to gastrointestinal complications occurring. In total, 17% of patients had a discontinuation of the ipilimumab sequence treatment due to AEs. 

While most AEs occurring during the nivolumab maintenance sequence were Grade ≤ 2, one Grade 5 AE occurred. Further investigation into that specific case revealed that the side effect occurred at the start of the nivolumab maintenance sequence, immediately after the end of the ipilimumab induction phase; thus, no conclusion can be drawn as to the individual contribution that these agents had in this specific case. Most Grade ≥ 3 were gastrointestinal disorders (colitis, including a case of ulcerative colitis, with the most common occurring symptom being diarrhea) and hepatic disorders (treatment-related hepatitis frequently manifesting itself through raised liver enzymes and high bilirubin levels). Most Grade ≤ 2 AEs consisted of gastroenterological, hepatic, and endocrine side effects with the most common gland disorders consisting of thyroiditis and subsequent sequelae, as shown in Table 2.

#### Subgroup Analyses

**Figure 3:**
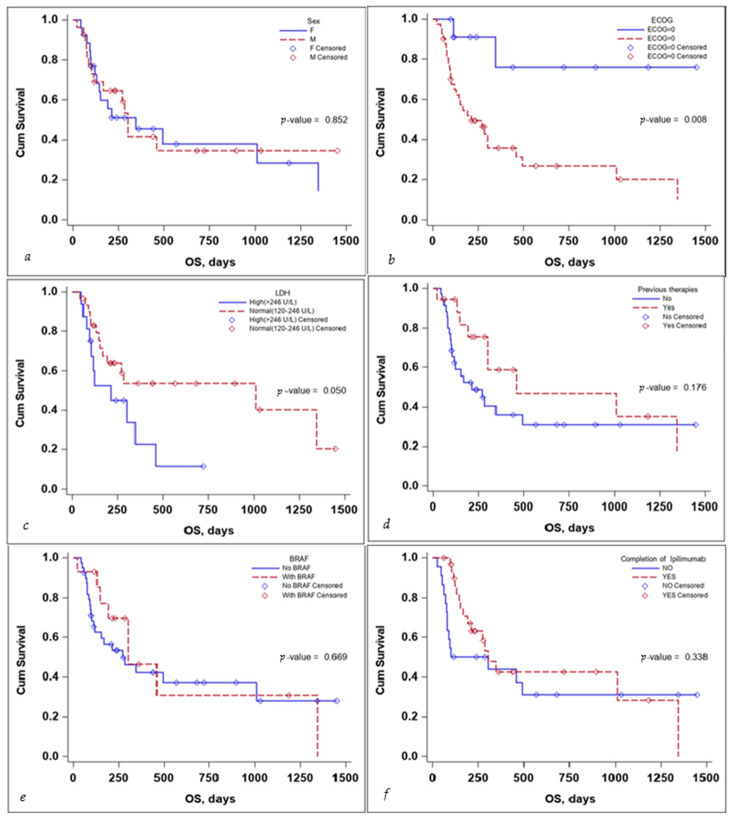
Kaplan–Meier curve for OS according to (**a**) sex, (**b**) ECOG, (**c**) LDH levels at the start, (**d**) previous treatment, (**e**) BRAF status, and (**f**) completion of treatment.

**Figure 4:**
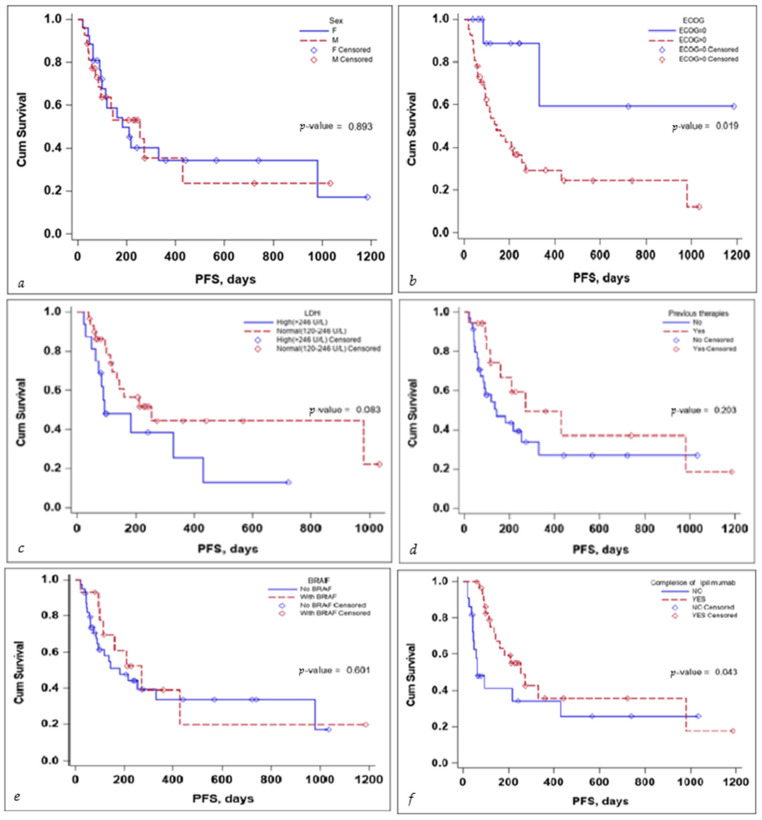
Kaplan–Meier curve for PFS according to (**a**) sex, (**b**) ECOG, (**c**) LDH levels at the start, (**d**) previous treatment, (**e**) BRAF status, and (**f**) completion of treatment.

Several subgroup analyses were performed to evaluate the impact that sex, ECOG status, BRAF mutation status, the completion of the ipilimumab sequence, previous therapies, and lactate dehydrogenase (LDH) levels would have on the OS and PFS of the patients, as seen in Figure 3 and Figure 4.

The sex of the patient did not influence the OS, with a median survival time of 346 days for females (95% CI: 130–1347) and 302 days for males (95% CI: 113–NA) with a *p*-value that did not reach statistical significance either on the log-rank test, *p*-value 0.852, or the multivariate Cox regression analysis, *p*-value 0.254, HR 95% CI: 1.74 (0.67–4.49), as seen in Table 3.

ECOG status and LDH levels both negatively influenced survival. A patient’s ECOG Performance Status Scale score of 0 had a positive impact on OS, with the Kaplan–Meier curve for OS as seen in Figure 3, the *p*-value on the log-rank test of 0.008, and a multivariate Cox regression *p*-value of 0.031 HR (95% CI): 0.16 (0.03–0.84).

While LDH levels just barely failed to meet the <0.05 significance value on the log-rank test: *p*-value = 0.050, the median survival time for patients with LDH values > 246 U/L was 212 days (95% CI: 94–346), while for patients with LDH values < 246 U/L was 1011 days (95% CI: 168–NA), the significance value was reached on the multivariate Cox regression analysis, with a *p*-value 0.030 HR (95% CI): 2.90(1.11–7.55), as seen in Table 3.

Receiving previous therapies, BRAF mutational status, and the completion of the ipilimumab induction sequence failed to reach statistical significance influencing OS, as seen in Table 3.

With regards to PFS, the sex of the patient and the BRAF mutation status did not seem to influence PFS in a significant manner. LDH levels seem to be associated with a lower PFS, but the significance value was reached only in the multivariate Cox regression analysis, HR (95% CI) 0.10 (0.02–0.58), with a *p*-value of 0.010—the scientific evidence does not allow for a definitive conclusion to be drawn due to the limited number of events in the group, as seen in Table 4. 

No definitive conclusion can be drawn with regard to the impact of previous therapies on PFS. While the log-rank test did not reach statistical significance (*p*-value 0.203), the number of events in the group was limited; thus, no definitive statement can be made in this regard. While completion of the ipilimumab induction sequence, 3 mg/kg for four cycles, tended to reach statistical significance, in the multivariate Cox regression analysis, HR (95% CI) 0.92 (0.09–9.77) the *p*-value 0.945 failed to reach statistical significance, as seen in Table 4.

## 4. Discussion

This study covers real-world data from 57 patients, out of which 4 were excluded due to a lack of complete medical data. We retrospectively analyzed the remaining 53 stage IV melanoma patients who received nivolumab and ipilimumab combination therapy with regards to survival data, incidence, and the severity of side effects based on real-world data collected from the Oncology Institute “Prof. Dr. Ion Chiricuță” Cluj-Napoca, Romania, and the Regional Institute of Oncology, Iași, Romania, between the years of 2019 up to the end of 2022.

LDH levels can be a useful tool in the initial diagnostic work-up for skin melanoma patients; as presented in our results, patients that have three or more sites of metastasis have a higher chance of having higher serum LDH levels, and our findings agree with the literature on this matter [9]. However, this marker has its limitations; for example, LDH levels do not associate as well as S-100B with the metabolic active tumor volume (evaluated via 18F-FDG PET/CT scans) [15]. There is also the problem of racial disparities between the expression of this marker [16,17] and other confounding factors and comorbidities that can cause high serum LDH levels, for example, other malignancies: lymphomas, pancreatic carcinoma, various liver metastases, etc., [18], and other diseases such as myocardial infarction, obstructive jaundice, acute hepatitis, etc., [18,19,20,21].

The OS values were lower than those reported in trials as the patients included in clinical trials are highly selected and most of them also include stage III patients who have a significantly better prognosis than stage IV patients. One solid piece of evidence for the efficacy of the combined ipilimumab and nivolumab regimen is the CheckMate 067 study. In the long-term results reported [22] for the ipilimumab–nivolumab combination, an OS of 49% is reported and a melanoma-specific survival (MSS) of 56%, with the median MSS not reached at a 6.5-year minimum follow-up. With a minimum follow-up of 7.5 years, the results remained consistent, with a median OS of 72.1 months for the combination therapy, 36.9 months for the nivolumab monotherapy, and 19.9 months for the ipilimumab monotherapy, the median MSS was as follows: not reached, 49.4 months, and 21.9 months. This study included patients of stage III/IV, previously untreated, unresectable with an ECOG performance status of 0–1, and excluded patients with ECOG ≥ 2, active brain metastases, uveal melanoma, and autoimmune diseases. When comparing the included population characteristics of the two studies, as in Table 5 [23], there are noticeable differences, especially with regard to the ECOG status, number of patients with brain metastases, and disease staging.

In comparison with results reported in the chemotherapy era of melanoma treatment, in which dacarbazine and paclitaxel + carboplatin regimens were staples of treatment in stage IV melanoma, a marked improvement can be noticed. Chemotherapy treatments in this scenario reported a median survival time of 7–9 months [24,25,26] and a one-year overall survival ranging between 30% and 65%, varying greatly based on the site of metastasis and LDH levels at diagnosis [27]. Meanwhile, our results reported an OS of 346 days with 17% of patients having CNS involvement and 30.2% of patients having high LDH levels at diagnosis.

Concerning side effects, most papers reported a varied frequency of side events: IMMUNED trial reported 71% (95% CI 57–82) Grade 3–4 adverse events with combined therapy (28.4 months median follow-up: IQR 17.7–36.8) [28]; CheckMate 915 [29] at a minimum follow-up of approximately 23.7 months reported 32.6% Grade 3–4 adverse events; Larkin et al. 2015 [30] reported treatment-related adverse events of Grade 3 or 4 occurred in 55% of the combination group (median follow-up ranged between 12.2 and 12.5 months across the three groups included). The frequency of treatment-related adverse events in our study was 45.3% with AEs ≥ 3 being reported in 26.4% with one Grade 5 event. Most ≥3 were gastrointestinal disorders (colitis, including a case of ulcerative colitis, with the most common occurring symptom being diarrhea) and hepatic disorders (treatment-related hepatitis frequently manifesting itself through raised liver enzymes and high bilirubin levels), as shown in Table 2.

Multivariate Cox regression analysis performed for LDH levels showed statistical significance in both OS and PFS; thus, LDH has both predictive and prognostic value. This serological marker also has the potential to be used as a complementary diagnostic tool as high LDH levels can be quite commonplace in a high-burden disease scenario. The result from our study is in line with current literature findings, as LDH levels are used as a stratification tool in M1 skin melanoma patients, as seen in the AJCC 8th edition [31].

This study has several limitations. One of the main limitations of this study is the retrospective nature of the analyzed data, thus providing a lower level of evidence when compared to prospective data. As mentioned in Section 1, melanoma in Romania has a 5-year prevalence of 25.04/100.000 inhabitants [1]; thus, the number of included subjects was limited, thus limiting the statistical significance of the data. The period of follow-up was also under 5 years, the earliest patients that could be identified with this type of treatment were from the early years of 2019—a period which coincides with the introduction date of the nivolumab + ipilimumab regimen in the metastatic setting for melanoma patients in Romania.

Future directions of research include extended research on this topic after a longer period of experience with such agents, analyzing other new agents used in this setting, and stratifying responses to therapies based on specific germinal or somatic mutations.

## 5. Conclusions

This study provides real-world insights into the survival data and safety profiles of combination therapy with anti-programmed death-1 antibodies (nivolumab) and anti-cytotoxic T-lymphocyte antigen-4 antibodies (ipilimumab), proving it to be an efficient treatment with a toxicity profile similar with other reporting. Also, LDH remains a serological marker useful in diagnostics as it is associated with a high disease burden, and it is also useful due to having additional prognostic and predictive value.

## Figures and Tables

**Table 1:** Descriptive statistics of patients’ characteristics.

Variable	Number of Patients (n = 53)
Age	
median (range)	54.1 (23–77)
Sex	
F	26 (49.1%)
M	27 (50.9%)
ECOG PS	
0	12 (22.6%)
1	33 (62.3%)
2	8 (15.1%)
Sites of metastatic disease (at the start of the treatment sequence)	
Bone	10 (18.9%)
CNS	9 (17.0%)
Hepatic	18 (34.0%)
Nonregional lymph nodes	30 (56.6%)
Lung	27 (50.9%)
Other soft tissues or organs	19 (35.8%)
LDH level	
High (>246 U/L)	16 (30.2%)
Normal (120–246 U/L)	30 (56.6%)
NA	7 (13.2%)
BRAF mutation status	
Mutated	23 (43.4%)
Wild type	25 (47.2%)
Unknown	5 (9.4%)
Sites of progression	
Bone	3 (5.7%)
CNS	4 (7.5%)
Hepatic	4 (7.5%)
Nonregional lymph nodes	3 (5.7%)
Local	1 (1.9%)
Lung	5 (9.4%)
Nul	41 (77.4%)
Other soft tissues or organs	4 (7.5%)

**Table 2:** Adverse events.

Adverse Event	Number of Patients (n, %)
Grade 1	
Hepatic disorder	3 (5.7%)
Thyroid	3 (5.7%)
Grade 2	
Dermatitis	2 (3.8%)
Gastrointestinal disorder	2 (3.8%)
Hematological disorder	1 (1.9%)
Hepatic disorder	1 (1.9%)
Pneumonitis	1 (1.9%)
Thyroid	4 (7.5%)
Grade 3	
Gastrointestinal disorder	4 (7.5%)
Hepatic disorder	1 (1.9%)
Grade 4	
Gastrointestinal disorder	3 (5.7%)
Hepatic disorder	5 (9.4%)
Grade 5	
Gastrointestinal disorder	1 (1.9%)

**Table 3:** Association of baseline characteristics with OS.

Variable	Kaplan–Meier Survival Analysis		Univariate Cox Regression Analysis		Multivariate Cox Regression Analysis	
	Median Survival Time (days) (95% CI)	*p*-Value (Log-Rank Test)	HR (95% CI)	*p*-Value	HR (95% CI)	*p*-Value
Sex (female vs. male)	346 (130–1347) 302 (113–NA)	0.852	1.11 (0.51–2.41)	0.791	1.74 (0.67–4.49)	0.254
ECOG (0 vs. >0)	NA (346–NA) 212 (120–460)	0.008	0.22 (0.05–0.92)	0.038	0.16 (0.03–0.84)	0.031
LDH (>246 vs. <246 U/L)	212 (94–346) 1011 (168–NA)	0.050	2.20 (0.93–5.20)	0.073	2.90 (1.11–7.55)	0.030
Previous therapies (none vs. yes)	212 (102–493) 460 (191–NA)	0.176	1.90 (0.79–4.56)	0.151	2.07 (0.23–18.81)	0.517
BRAF (none vs. present)	271 (113–1011) 303 (144–1347)	0.669	1.46 (0.58–3.64)	0.419	1.65 (0.15–18.41)	0.686
Completion of ipilimumab (no vs. yes)	200 (73–NA) 303 (191–1347)	0.338	1.52 (0.70–3.31)	0.292	1.25 (0.50–3.14)	0.631

Note: Due to the small number of events in a group, estimates of the median and/or its upper confidence interval limit are not available and are marked with NA.

**Table 4:** Association of baseline characteristics with PFS.

Variable	Kaplan-Meier Survival Analysis		Univariate Cox Regression Analysis		Multivariate Cox Regression Analysis	
	Median Survival Time (days) (95% CI)	*p*-Value (Log-Rank Test)	HR (95% CI)	*p*-Value	HR (95% CI)	*p*-Value
Sex (female vs. male)	182 (99–981) 253 (83–NA)	0.893	0.95 (0.46–1.96)	0.893	0.98 (0.94–1.02)	0.322
ECOG (0 vs. >0)	NA (83–NA) 144 (93–253)	0.019	0.21 (0.05–0.89)	0.034	1.26 (0.53–3.03)	0.601
LDH (>246 vs. <246 U/L)	93 (63–430) 253 (119–NA)	0.083	2.02 (0.90–4.52)	0.089	0.10 (0.02–0.58)	0.010
Previous therapies (none vs. yes)	144 (72–330) 272 (113–NA)	0.203	1.66 (0.75–3.64)	0.209	3.35 (1.31–8.55)	0.012
BRAF (none vs. present)	182 (83–981) 272 (99–NA)	0.601	1.24 (0.55–2.79)	0.602	2.10 (0.23–18.90)	0.509
Completion of ipilimumab (no vs. yes)	63 (43–430) 253 (144–981)	0.043	2.09 (1.01–4.33)	0.048	0.92 (0.09–9.77)	0.945

Note: Due to the small number of events in a group, estimates of the median and/or its upper confidence interval limit are not available and are marked with NA.

**Table 5:** Baseline characteristics comparison between the patients included in the CheckMate 067 study and the patients included in this study [22].

CheckMate 067	Baseline Characteristics of This Study
Mean age—60 y	Mean age—54.1 y
Female—35.4%	Female—49.1%
ECOG 0—73.2%	ECOG 0—22.6%
ECOG 1—26.6%	ECOG 1—62.3%
ECOG 2—0.1%	ECOG 2–15.1%
LDH levels ≤ ULN—62.3%	LDH levels ≤ ULN—56.6%
No brain metastases—96.4%	No brain metastases—83%
Wild-type BRAF—68.5%	Wild-type BRAF—47.2%
Disease stages included—III and IV	Disease stage included—IV

## Data Availability

Data are contained within the article.
